# The effect of inspiratory rise time on mechanical power calculations in pressure control ventilation: dynamic approach

**DOI:** 10.1186/s40635-023-00584-6

**Published:** 2023-12-20

**Authors:** Özlem Acicbe, Canan Yazıcı Özgür, Payam Rahimi, Emral Canan, Sinan Aşar, Zafer Çukurova

**Affiliations:** 1 Department of Anesthesiology and Reanimation, Şişli Hamidiye Etfal Training and Research Hospital, University of Health Sciences, Istanbul, Turkey; 2 Department of Anesthesiology and Reanimation, Bakırköy Dr. Sadi Konuk Training and Research Hospital, University of Health Sciences, Istanbul, Turkey

**Keywords:** Mechanical power, PCV, ARDS, VILI

## Abstract

**Background:**

Mechanical power may serve as a valuable parameter for predicting ventilation-induced injury in mechanically ventilated patients. Over time, several equations have been developed to calculate power in both volume control ventilation (VCV) and pressure control ventilation (PCV). Among these equations, the linear model mechanical power equation (MP_LM_) closely approximates the reference method when applied in PCV. The dynamic mechanical power equation (MP_dyn_) computes power by utilizing the ventilatory work of breathing parameter (WOB_v_), which is automatically measured by the mechanical ventilator. In our study, conducted in patients with Covid-19 Acute Respiratory Distress Syndrome (C-ARDS), we calculated mechanical power using both the MP_LM_ and MP_dyn_ equations, employing different inspiratory rise times (T_slope_) at intervals of 5%, ranging from 5 to 20% and compared the obtained results.

**Results:**

In our analysis, we used univariate linear regression at both I:E ratios of 1:2 and 1:1, considering all T_slope_ values. These analyses revealed that the MP_dyn_ and MP_LM_ equations exhibited strong correlations, with *R*^2^ values exceeding 0.96. Furthermore, our Bland–Altman analysis, which compared the power values derived from the MP_dyn_ and MP_LM_ equations for patient averages and all measurements, revealed a mean difference of −0.42 ± 0.41 J/min (equivalent to 2.6% ± 2.3%, *p* < 0.0001) and −0.39 ± 0.57 J/min (equivalent to 3.6% ± 3.5%, *p* < 0.0001), respectively. While there was a statistically significant difference between the equations in both absolute value and relative proportion, this difference was not considered clinically relevant. Additionally, we observed that each 5% increase in T_slope_ time corresponded to a decrease in mechanical power values by approximately 1 J/min.

**Conclusions:**

The differences between mechanical power values calculated using the MP_dyn_ and MP_LM_ equations at various T_slope_ durations were determined to lack clinical significance. Consequently, for practical and continuous mechanical power estimation in Pressure-Controlled Ventilation (PCV) mode, the MP_dyn_ equation presents itself as a viable option. It is important to note that as T_slope_ times increased, the calculated mechanical power exhibited a clinically relevant decrease.

**Supplementary Information:**

The online version contains supplementary material available at 10.1186/s40635-023-00584-6.

## Introduction

Mechanical power (MP) is an emerging parameter that combines all respiratory variables contributing to ventilator-induced lung injury (VILI), enabling predictions at the bedside (1, 2). Various equations, differing in accuracy and complexity, have been introduced to compute MP during both volume control ventilation (VCV) and pressure control ventilation (PCV) (see Additional file 1: Table S1) (2–5). However, the precision and practicality of power equations developed for PCV mode remain subjects of ongoing debate (1, 6–9). This stems from the mathematical complexity of PCV equations and the need for inspiratory resistance, a parameter not easily accessible at the bedside (10). As a result, the integration of mechanical power into clinical practice has been delayed.

To address this challenge, we introduced and published the dynamic mechanical power equation (MP_dyn_) as one of the methods for calculating mechanical power (3). More recently, Trinkle et al. presented a simplified equation (Linear Model Mechanical Power Equation, MP_LM_) for calculating mechanical power in PCV mode in their latest publication. When compared to the gold standard geometric method, the MP_LM_ equation demonstrated superior performance, with an 8% bias, outperforming the Becher comprehensive equation (PCV_slope_, 10% bias) and the Van der Meijden equation (MP_pcv_, 16.5% bias). As a result, we have chosen the MP_LM_ equation as the reference method for our study. Since T_slope_ is a key factor in this equation, our aim is to investigate the impact of varying T_slope_ values on mechanical power.

In this study, we calculate mechanical power using both the MP_LM_ and MP_dyn_ equations in patients with Covid-19-related Acute Respiratory Distress Syndrome (C-ARDS) undergoing pressure control ventilation (PCV) with Inspiration/Expiration ratios (I:E) of 1:2 and 1:1. We also assess the influence of different T_slope_ durations (5%, 10%, 15%, and 20%) on these equations. Furthermore, we performed a comprehensive comparison of all equations under two different I:E ratios and across the four T_slope_ durations.

## Material and methods

### Study design and data collection

This study was performed in the general intensive care unit of the University of Health Sciences, Bakırköy Dr. Sadi Konuk Training and Research Hospital, between June 10, 2022, and January 15, 2023. The study included 38 patients diagnosed with Covid-19-related Acute Respiratory Distress Syndrome (C-ARDS) who met the following criteria: deep sedation, paralysis, and mechanical ventilation in pressure control ventilation (PCV) mode. Patients with chronic obstructive pulmonary disease (COPD) and heart failure, pregnant individuals, patients with tube thoracostomy, and hemodynamically unstable patients were excluded from the study. A flowchart describing the patient selection process is provided in Additional file 1: Figure S1. Clinical data, including Sequential Organ Failure Assessment (SOFA) scores, Acute Physiology and Chronic Health Evaluation-II (APACHE II) scores, age, gender, height, predicted body weight (PBW), ideal weight, and body mass index (BMI), were retrieved from the hospital's database using SQL queries and transferred to Microsoft Excel for analysis.

All patients were ventilated using a Maquet Servo-U ventilator (Gothenburg, Sweden) in PCV mode. Patient data were recorded on the ventilator at a scanning rate of 20 mm/s. Patients were intubated with a 7.5 mm endotracheal tube and were provided humidification using a heat and moisture exchange (HME) filter (GIBECK Humid-Vent, UK). Active humidification systems were not employed during the study.

Inspiratory pressure change (∆P_insp_) was adjusted to achieve a tidal volume of 4–6 ml/kg based on Predicted Body Weight (PBW). Positive end-expiratory pressure (PEEP) was set between 8 and 12 cmH_2_O, and the Fraction of Inspired Oxygen (FiO_2_) was adjusted to range from 40 to 80%, targeting an oxygen saturation (SpO_2_) of 88–92%. The parameter T_slope_ was varied between 5 and 20% (5%, 10%, 15%, and 20%) under two different Inspiration/Expiration (I:E) ratios, 1:2 and 1:1, with 5% increments every 20 min. To assess the presence of intrinsic PEEP, total PEEP was measured by performing an expiratory hold maneuver at each change in T_slope_ setting.

Mechanical ventilator and respiratory parameters of all patients were recorded with 'ImdSoft-Metavision/QlinICU Clinical Decision Support System (Israel). Data from mechanical ventilators, including end-inspiratory pressure (P_peak_), ∆P_insp_, PEEP, mean airway pressure (P_mean_), respiratory rate (RR), expiratory tidal volume (TVe), inspiratory time (T_insp_), compliance (C, calculated automatically by the ventilator: ∆V / P_peak_—PEEP), work of breathing ventilator (WOBv), I:E ratio, end-tidal carbon dioxide (etCO_2_), SpO_2_, and arterial blood gas values at admission (pH, PaO_2_, HCO_3_, PaCO_2_, BE), were extracted from the data pool using SQL queries. Statistical analyses were performed after calculating patient averages using Microsoft Excel.

If the end expiratory flow (V̇_ee_) is close to zero, it is considered that there is no intrinsic PEEP (11). V̇_ee_ flow values of 6080 min (38 × 4x20 = 3040 min for each I:E ratio) obtained in I:E 1:2 and I:E 1:1 ratios of 38 patients included in the study were measured close to zero (0.012 vs 0.035 L/s, respectively). See supplementary data for mean, confidence interval and P values of V̇_ee_ flow values measured at T_slope_ 5%, 10%, 15% and 20% (Additional file 1: Table S4). In addition, there was no clinically significant difference between the total PEEP obtained with the expiratory hold maneuver performed at the beginning of each 5% T_slope_ (5%-20%) change and the PEEP values set for all measurements (9.07 vs 9.22 cmH_2_O). Therefore, it was assumed that the patients included in the study did not have intrinsic PEEP.

Respiratory mechanics were obtained for each patient for a total of 80 min (80 min for I:E = 1:2 and 80 min for 1:1) with measurements of 20 min at each T_slope_ time, (Respiratory rate (RR), PEEP, expiratory tidal volume (TVe), ∆P_insp_, T_slope_, WOBv, expiratory velocity (Vee), FiO_2_, SpO_2_ and End-tidal carbon dioxide (EtCO_2_)). No adverse events observed requiring changes in these parameters (∆P_insp_, PEEP, RR, and FiO_2_) during the study. ∆P_insp_, PEEP, RR, and FiO_2_ settings were constant throughout the study period in each patient. SPO_2_, etCO_2_ and all hemodynamic parameters and other vital signs of the patients were monitored at the bedside.

### Diagnosis of C-ARDS

The diagnosis of COVID-19 in all 38 patients was confirmed through a combination of chest computed tomography (CT) imaging and nasal swab sample with Polymerase Chain Reaction (PCR) testing using the Bio-Speedy Covid-19 RT-qPCR detection Kit (Bioeksen, Turkey). Furthermore, the diagnosis of Acute Respiratory Distress Syndrome (ARDS) was established following the criteria outlined in the Berlin definition (12). Among the 38 patients included in the study, three patients were classified as having mild ARDS, 20 had moderate ARDS, and 15 were diagnosed with severe ARDS.

### Calculation of the dynamic mechanical power (MP_dyn_)

WOBv is the amount of energy spent to ventilate one liter of gas and is expressed as J/L (16, 17). In this study, WOBv values were obtained from mechanical ventilators (Maquet Servo-U, Sweden), which is calculated as described Cabello and Mancebo (16) using following quation:

$${\text{WOB}}=\int {\text{Pressure}} \times \text{Volume}$$

Then, WOBv values were dived by the tidal volume (17), which is calculated by mechanical ventilator, expressed as J/L.

To calculate mechanical power (MP_dyn_), we multiplied the minute volume (MVe) by the WOBv values (3), assuming expiratory valve is inactive.

$${\text{MP}}_{{{\text{dyn}}}} \, = \,{\text{WOB}}_{{\text{v}}} \, \times \,{\text{MV}}_{{\text{e}}}$$

With the contribution of PEEP, (0.098 × RR x TVe x PEEP) (2) the equation is formulated as follows:

$${\text{MP}}_{{{\text{dyn}}}} \, = \,{\text{MV}}_{{\text{e}}} \, \times \,\left[ {\left( {{\text{WOBv}}} \right)\, + \,\left( {{\text{PEEP}}\, \times \,0.0{98}} \right)} \right]$$

MVe is the expiratory minute volume (L/min), WOBv is the Work of Breathing ventilator (J/L), PEEP is the Positive End Expiratory Pressure (cmH_2_O), and 0.098 is the transformation factor.

### Calculation of the linear model mechanical power (MP_LM_)

Mechanical power calculation was performed using the Linear Model mechanical power equation (MP_LM_) by Trinkle et al.

$${\text{MP}}_{{{\text{LM}}}} = 0.0{98}\, \times \,{\text{RR}}\, \times \,\left[ {{\text{TVe}}\, \times \,\left( {{\text{PEEP}}\, + \,\Delta {\text{P}}_{{{\text{insp}}}} } \right)\, - \,0.{15}\, \times \,\Delta {\text{P}}_{{{\text{insp}}}}^{{2}} \, \times \,{\text{T}}_{{{\text{slope}}}} /{\text{Ri}}} \right]$$

RR is the Respiratory rate, ∆P_insp_ is the Inspiratory airway pressure change (cmH_2_O), PEEP is the Positive End Expiratory Pressure (cmH_2_O), TVe is the Expiratory tidal volume (L), Ri is the Inspiratory resistance (cmH_2_O/L/s), T_slope_  is the Inspiratory rise time (sc), and 0.098 is the transformation factor.

In PCV mode, inspiratory resistance (Ri) used in mechanical power calculation with MP_LM_ and MP_slope_ equations can only be measured with the least square fit method (LSF) in mechanical ventilators. Since a fixed T_pause_ time is set in volume control ventilation, the inspiratory resistance is continuously measured automatically by the mechanical ventilator. In ARDS patients, the median inspiratory resistance value in VCV mode is considered to be 10 cmH_2_O s/L (4, 15, 16). In this way, practical alternatives to the complex VCV equation have been developed (4, 17). This approach in VCV mode can also be applied to PCV mode.

Arnal JM et al. calculated the median inspiratory resistance value with LSF as 15 cmH_2_O s/L in ARDS patients ventilated in PCV mode (the same value is valid for adult non-ARDS patients without COPD) (10, 18).

In our study, mechanical power was calculated at T_slope_ 5%, 10%, 15% and 20% with I:E ratios 1:2 and 1:1 by using the median Ri value of 15 cmH_2_O s/L instead of inspiratory resistance used in MP_LM_ equation. Among the mechanical power equations developed for PCV mode, the MP_LM_ equation was preferred as the reference equation in this study because it calculates the closest values (bias 8%) to the geometric method, which is accepted as the gold standard method in power calculations (1). With a median value of 15 cmH_2_O s/L of inspiratory resistance, both MP_LM_ and MP_dyn_ equations were compared with each other in T_slope_ of 5%, 10%, 15% and 20%.

### Statistical methodology

To compare the mechanical power values obtained from different equations, univariate linear regression was employed to analyze pairs of equations. Bland–Altman analysis, with a subsequent simple t-test for post hoc analysis, was utilized to further assess the similarity or divergence between these methods. Multiple comparisons of respiratory mechanics, including mechanical power, obtained at T_slope_ values of 5%, 10%, 15%, and 20%, were conducted using a repeated measures Multivariate Analysis of Variance (MANOVA) approach. Post hoc analyses were performed using the Tukel test. All statistical analyses were carried out using GraphPad Prism version 9.3.1.471 (Serial Number: GPS-2345356-TCTS-A6818).

Measurements were made for 160 min for each patient and 6080 min for a total of 38 patients. Data were saved in ImdSoftMetavision/QlinICU Clinical Decision Support Software. Statistical analyzes were performed on the basis of patient averages and data (in minutes). Subgroup analyses were performed based on both Inspiration/Expiration (I:E) ratios and T_slope_. To establish the required sample size, a preliminary study was conducted involving 5 patients. The primary outcome measure was the difference between the MP_dyn_ and MP_LM_ equations. The mean difference between the equations was approximately 0.35 J/min, with a standard deviation of approximately 0.5 J/min. Power analysis was then conducted, considering an 85% power difference with a standard error of 0.05. Based on these parameters, the sample size for the study was determined to be 38 patients when the maximum mean difference and standard deviation of the difference between methods were approximately 0.5 ± 1.0 J/min, as calculated using G*power version 3.

## Results

The characteristics of 38 ARDS patients, including mean and standard deviation values for demographic data, scores, arterial blood gases and respiratory mechanics, providing a comprehensive review of these parameters are displayed in Table 1.

**Table 1:** Patient characteristics

Patients characteristics (n:38)	Means and sd
Gender, female (%)	16 (42%)
Height (m)	1,71 ± 0,08
Weight (kg)	81,9 ± 11,7
Body mass index (kg/m^2^)	28,2 ± 3,7
Predicted body weight (kg)	64,4 ± 9,6
Age (years)	60,7 ± 17,1
pH	7,29 ± 0,13
PaCO_2_ (mmHg)	56 ± 16
PaO_2_ (mmHg)	92 ± 24
PaO_2_/FiO_2_ (mmHg/%)	152 ± 52
FiO_2_ (%)	64 ± 15
HCO_3_ (meq/dL)	22,9 ± 4,9
BE (meq/dL)	−1,9 ± 6,2
∆P_insp_ (cmH_2_O)	16,1 ± 3,0
PEEP (cmH_2_O)	9,7 ± 2,0
TV_e_/PBW (ml/kg)	7,2 ± 1,4
P_peak_ (cmH_2_O)	25,8 ± 3,0
Minute volume (L/minute)	6,9 ± 1,6
Compliance (ml/cmH_2_O)	29,0 ± 7,0
WOBv (J/L)	1,3 ± 0,23
Vee (ml/s)	0,024 ± 0,016
APACHE II score first and last	23 ± 9 and 26 ± 11
Expected mortality (%)	48 ± 25
ICU mortality	22 (58%)
SOFA score first and last	10 ± 4 and 12 ± 6
Length of stay in ICU (days)	16,8 ± 8,2
Invasive mechanical ventilation time (day)	15,0 ± 7,4

Mechanical power was calculated for four different T_slope_ values (5%, 10%, 15%, and 20%) under two different Inspiration/Expiration (I:E) ratios (1:2 and 1:1) using both the MP_LM_ equation and the dynamic mechanical power equation (MP_dyn_) in the cohort of 38 patients with Covid-19-related Acute Respiratory Distress Syndrome (C-ARDS). These calculations were then subjected to analysis through univariate linear regression and Bland–Altman analysis to elucidate the relationships and differences between the two equations.

### Univariate linear regression and Bland–Altman analysis

**Fig. 1:**
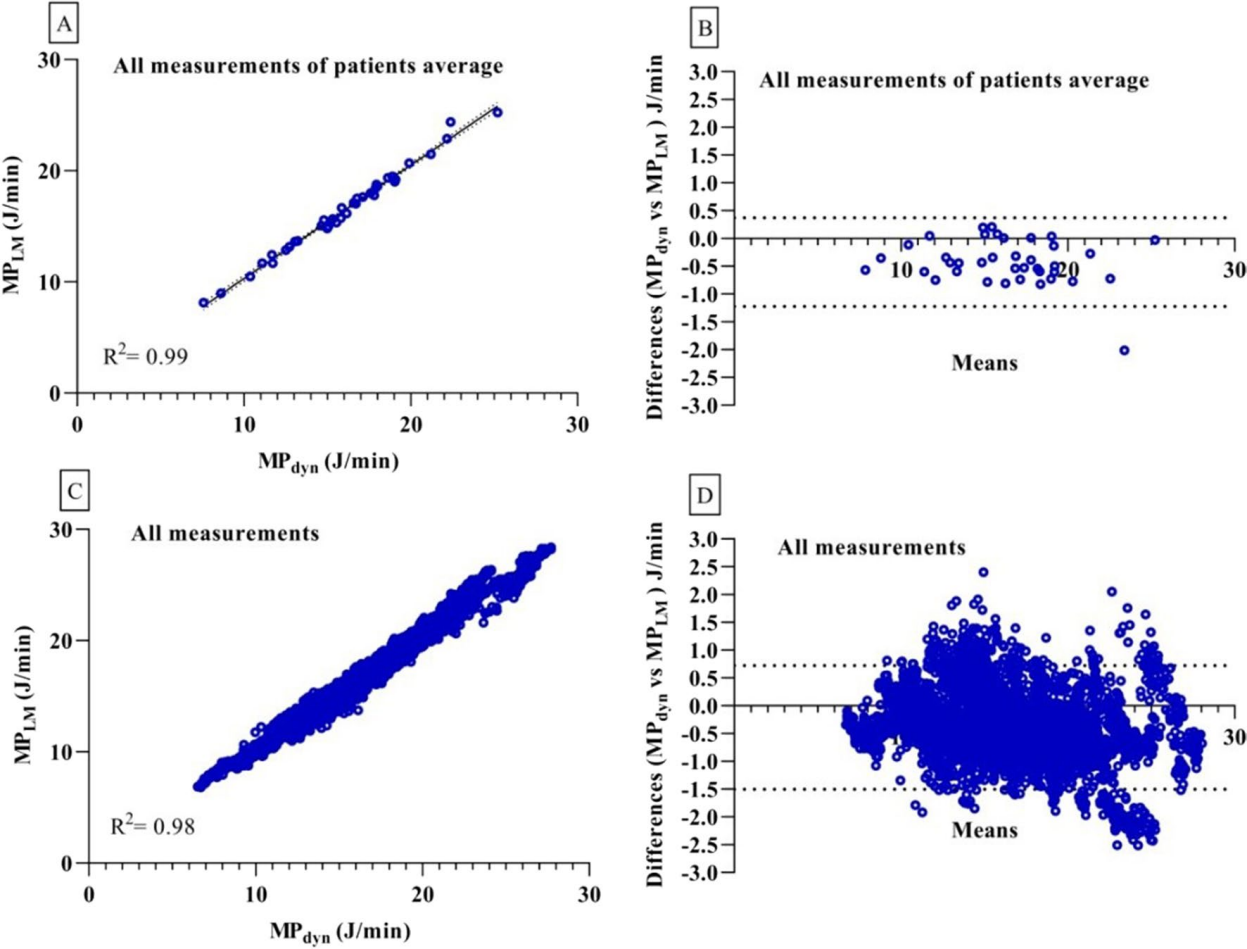
Univariate lineer regression and Bland–Altman based analyses on mechanical power of all patients average and all measurements. **A**. MP_LM_ (y-axis)—is plotted as function of the MP_dyn_ equation (x-axis), **B**. MP_dyn_- MP_LM_ corresponding Bland–Altman plots. C. MP_LM_ (y-axis)—is plotted as function of the MP_dyn_ equation (x-axis), D. MP_dyn_- MP_LM_ corresponding Bland–Altman plots

In the univariate linear regression analysis was performed on the mean power values of all 38 patients, encompassing 6080 min of data, both the MP_dyn_ and MP_LM_ equations displayed significant correlations (*y* = 1.0 ± 0.018, *R*^2^ = 0.99, and *y* = 1.0 ± 0.002, *R*^2^ = 0.98, respectively, *P* < 0.0001, Fig. 1A and C).

Bland–Altman analysis based on the mean power values of all patients and all measurements revealed mean differences and standard deviations of bias values as −0.42 ± 0.41 J/min (2.6% ± 2.3%, *P* < 0.0001) and −0.39 ± 0.57 J/min (3.6% ± 3.5%, *P* < 0.0001), respectively, indicating statistical significance (Fig. 1B and D).

Patient mean power values calculated with MP_dyn_ and MP_LM_ equations at T_slope_ 5%, 10%, 15%, and 20% for an Inspiration/Expiration (I:E) ratio of 1:2 were correlated in univariate linear regression analysis (*y* = 1.0 ± 0.015, *R*^2^ = 0.99; *y* = 1.0 ± 0.018, *R*^2^ = 0.99; *y* = 1.0 ± 0.035, *R*^2^ = 0.96; *y* = 1.0 ± 0.027, *R*^2^ = 0.98, respectively; *P* < 0.0001, see Additional file 1: Figure S3A, B, C, D).

**Fig. 2:**
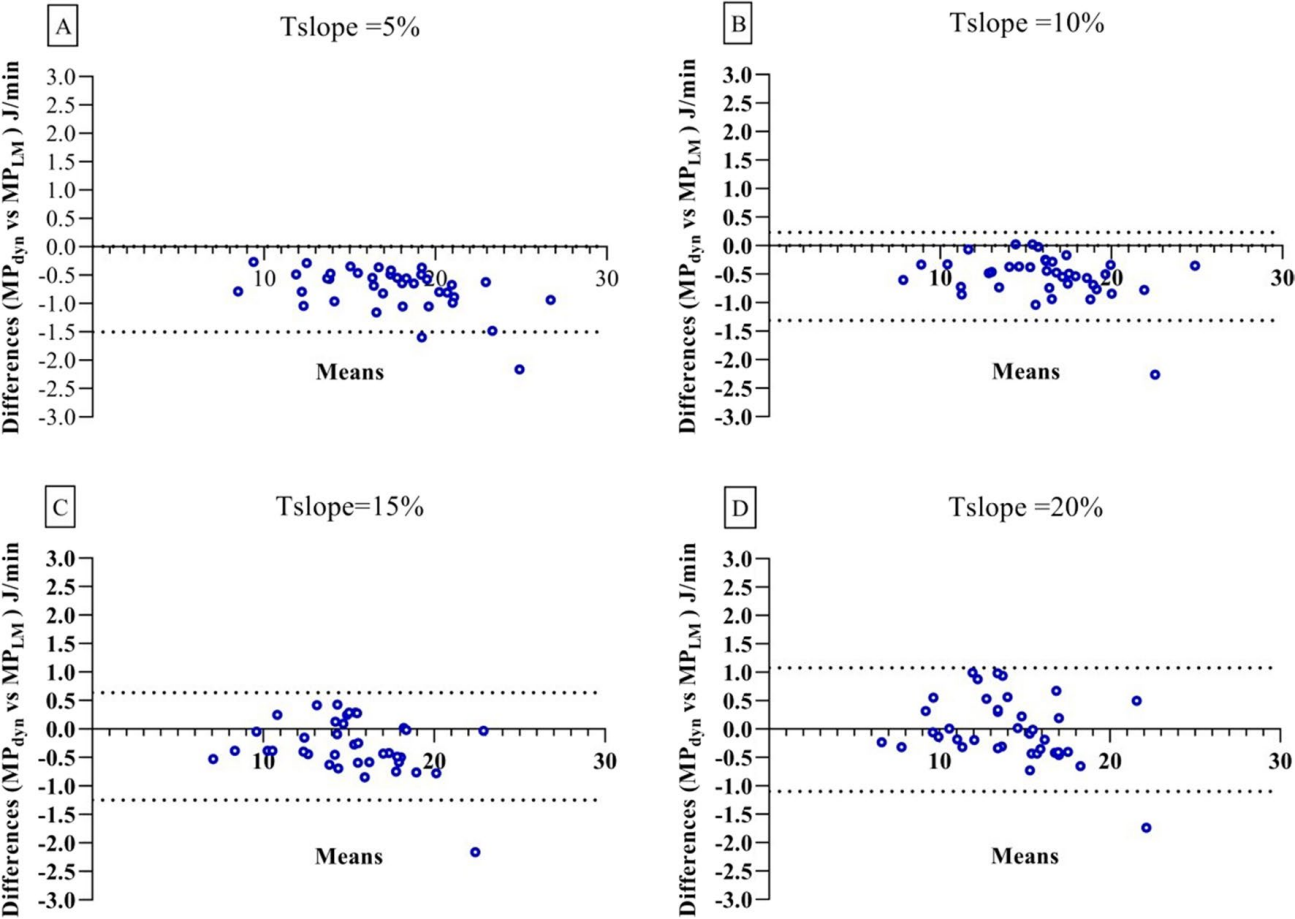
Bland–Altman based analyses on mechanical power of all patients average. For I:E 1:2 T_slope_ 5%: **A** MP_dyn_- MP_LM_ corresponding Bland–Altman plots. For I:E 1:2 T_slope_ 10%: **B** MP_dyn_- MP_LM_ corresponding Bland–Altman plots. For I:E 1:2 T_slope_ 15%: **C** MP_dyn_- MP_LM_ corresponding Bland–Altman plots. For I:E 1:2 T_slope_ 20%: **D** MP_dyn_- MP_LM_ corresponding Bland–Altman plots

In T_slope_ 5%, 10%, and 15% Bland–Altman analysis, mean differences and standard deviations of bias values were calculated as −0.75 ± 0.39 J/min (4.2% ± 1.9%, *P* < 0.0001), −0.54 ± 0.39 J/min (3.3% ± 2.1%, *P* < 0.0001), −0.31 ± 0.48 J/min (1.9% ± 2.8%, *P* = 0.0004), respectively, all of which were statistically significant (Fig. 2A, B, C). The mean difference and standard deviation of bias values for T_slope_ 20% were calculated as −0.01 ± 0.55 J/min (0.2% ± 3.8%) and were not statistically significant (*P* = 0.91, Fig. 2D).

Patient mean power values calculated with MP_dyn_ and MP_LM_ equations at T_slope_ 5%, 10%, 15%, and 20% for an I:E ratio of 1:1 were correlated in univariate linear regression analysis (*y* = 1.0 ± 0.015, *R*^2^ = 0.99; *y* = 1.0 ± 0.014, *R*^2^ = 0.99; *y* = 1.0 ± 0.020, *R*^2^ = 0.99; *y* = 0.99 ± 0.026, *R*^2^ = 0.98, respectively; *P* < 0.0001, see Additional file 1: Figure S4A, B, C, D).

**Fig. 3:**
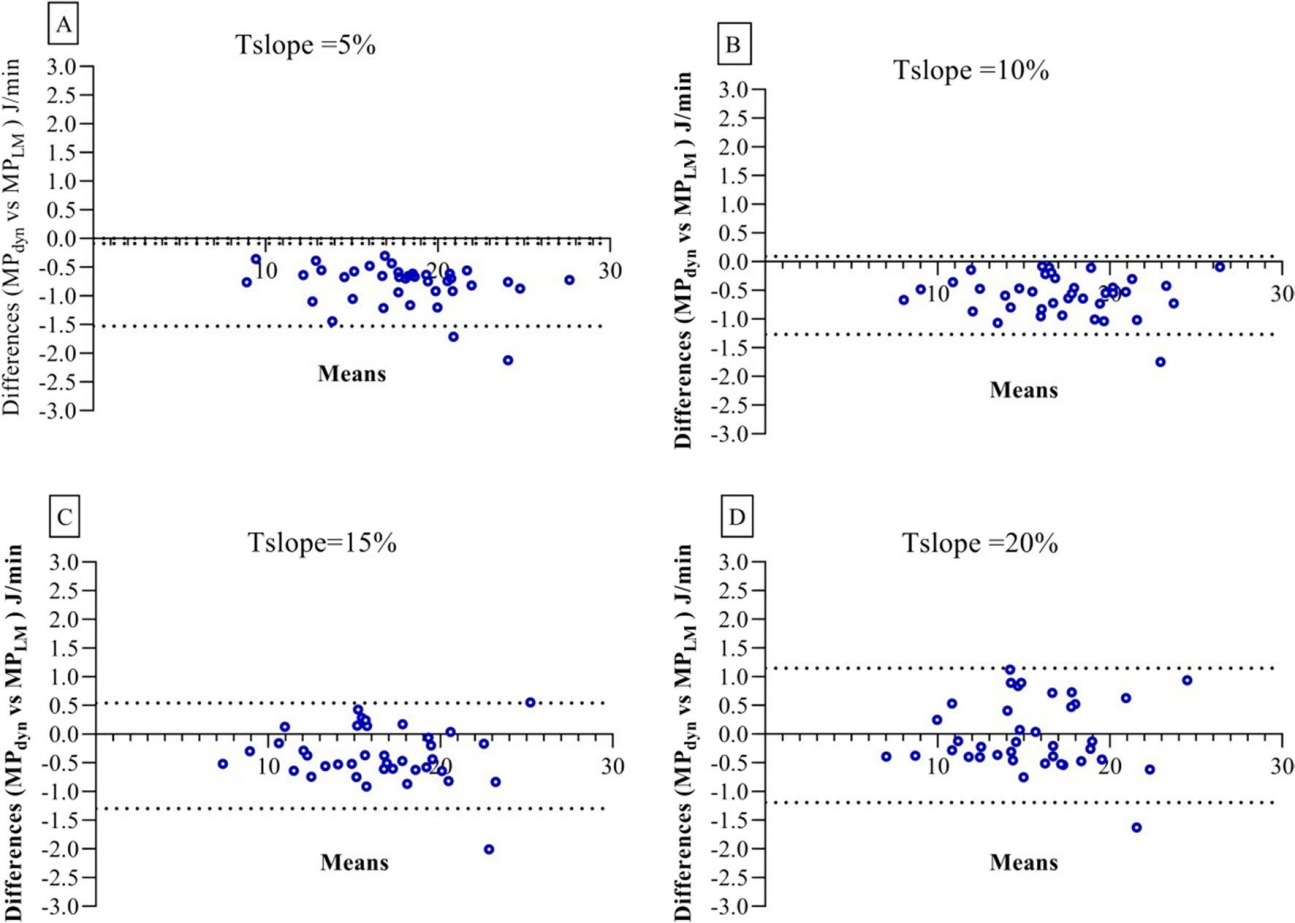
Bland–Altman based analyses on mechanical power of all patients average. For I:E 1:1 T_slope_ 5%: **A** MP_dyn_- MP_LM_ corresponding Bland–Altman plots. For I:E 1:1 T_slope_ 10%: **B** MP_dyn_- MP_LM_ corresponding Bland–Altman plots. For I:E 1:1 T_slope_ 15%: **C** MP_dyn_- MP_LM_ corresponding Bland–Altman plots. For I:E 1:1 T_slope_ 20%: **D** MP_dyn_- MP_LM_ corresponding Bland–Altman plots

In T_slope_ 5%, 10%, and 15% Bland–Altman analysis, mean differences and standard deviations of bias values were calculated as −0.81 ± 0.37 J/min (4.5% ± 2.0%, *P* < 0.0001), −0.59 ± 0.35 J/min (3.5% ± 2.1%, *P* < 0.0001), −0.38 ± 0.47 J/min (2.3% ± 2.6%, *P* < 0.0001), respectively, all of which were statistically significant (Fig. 3A, B, C). The mean difference and standard deviation of bias values for T_slope_ 20% were calculated as −0.02 ± 0.60 J/min (0.2% ± 3.8%) and were not statistically significant (*P* = 0.81, Fig. 3D).

The effect of 5% increase in T_slope_ on the mechanical power values calculated with MP_LM_ and MP_dyn_ are represented in Additional file 1: Table S2.

Additional file 1: Table S3 displays a comparison of respiratory mechanics, encompassing ∆P_insp_, PEEP, MVe, and WOBv, for T_slope_ values of 5%, 10%, 15%, and 20% between MP_LM_ and MP_dyn_. Additionally, Additional file 1: Table S4 presents patient averages of RR, TVe, C, T_insp_, end-expiratory velocity (Vee), FiO_2_, SpO_2_, and etCO_2_ at T_slope_ 5%, 10%, 15%, and 20% for I:E ratios of 1:2 and 1:1.

## Discussion

Monitoring mechanical power (MP), a parameter that combines key variables involved in ventilator-induced lung injury (VILI), holds promise as a valuable tool in guiding lung-protective ventilation for critically ill patients (19). However, the practical implementation of MP calculations, particularly in pressure control ventilation (PCV), has presented challenges (10). In this study, the dynamic mechanical power (MP_dyn_) equation, which calculates MP in PCV mode without the need for inspiratory resistance measurement, was compared with the Linear Model mechanical power equation (MP_LM_) (1) in Covid-19-related Acute Respiratory Distress Syndrome (C-ARDS) patients. The study assessed the impact of different inspiratory rise times (T_slope_) on MP values.

The results of this study revealed that MP values calculated using the MP_dyn_ and MP_LM_ equations were highly correlated, indicating that both equations can provide consistent estimations of MP. The most significant differences between the two equations were observed at T_slope_ 5%, with MP_dyn_ consistently showing lower values compared to MP_LM_. However, these differences diminished as T_slope_ increased, and no statistically significant difference was found at T_slope_ 20%. Interestingly, while the differences between the two equations were statistically significant at T_slope_ 5%, these differences were not considered clinically relevant. This finding suggests that despite the statistical variance, both equations produce MP values that are practically equivalent for clinical decision-making. We also observed that increasing T_slope_ resulted in a decrease in MP values calculated by both equations. This decrease in MP with longer T_slope_ times can be explained by the fact that slower inspiratory gas flow at the beginning of inspiration applies less energy to the respiratory system. Importantly, this decrease in MP may be beneficial, as high energy levels have been linked to lung damage in both experimental and clinical studies. It is known that MP values exceeding certain threshold values (12 J/min for ARDS and 17 J/min for non-ARDS patients) can lead to significant lung injury. Thus, reducing MP, especially in ARDS patients, is desirable to minimize the risk of VILI.

The study's findings are consistent with the notion that flow patterns and rates are crucial in ARDS patients. In PCV, MP is concentrated at the beginning of inspiration, which can be problematic, whereas in volume control ventilation (VCV), MP is distributed more evenly throughout inspiration. Increasing T_slope_ to slow the gas flow at the beginning of inspiration can reduce the concentration of power and minimize the risk of damage to the lungs.

In conclusion, this study underscores the practicality and clinical relevance of calculating MP using both the MP_dyn_ and MP_LM_ equations in PCV mode. While differences in MP values were observed at lower T_slope_ settings, these differences were not considered clinically significant. The study also highlights the potential benefits of adjusting T_slope_ to lower MP values, thereby reducing the risk of VILI. Additionally, the MP_dyn_ equation offers a convenient option for continuously and in real time calculating MP in PCV mode without the need for inspiratory resistance measurements, which could prove valuable in clinical practice.

**Fig. 4:**
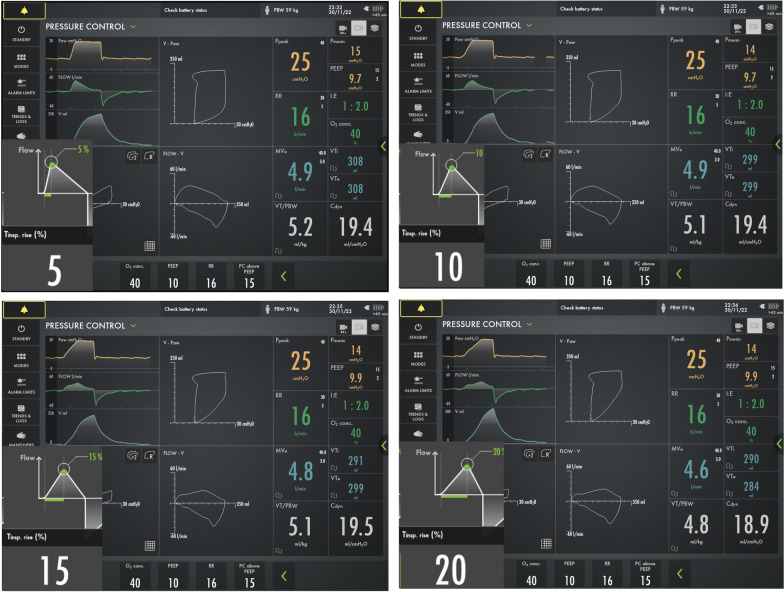
Dynamic pressure–volume loop (P–V), pressure- time, flow- time and volume- time graphics of I:E 1:2 and T_slope_ 5%, 10%, 15% and 20% in PCV mode (obtained with screen shots of the Servo-U mechanical ventilator)

However, without altering the fundamental respiratory parameters (∆P_insp_, RR, and PEEP) that constitute mechanical power, each 5% increase in T_slope_ led to an approximate 1 J/min reduction in the mechanical power values calculated using both the MP_LM_ and MP_dyn_ equations. Consequently, an approximate 3 J/min difference was observed between the power values at T_slope_ 5% and 20%. Since all the respiratory mechanics contributing to power remained constant (no difference in MV_e_ at I:E 1:1, and a clinically insignificant decrease at I:E 1:2), no changes were expected in the static and dynamic elastic components of power (PEEP and driving pressure). The reason behind the decrease in mechanical power was attributed to the constriction of the area forming the resistive component of the P–V loop as T_slope_ increased (refer to Fig. 4 for I:E 1.2 and Additional file 1: Figure S2 for 1:1).

Trinkle et al. (1) reported that mechanical power decreases as T_slope_ increases, and our study is in agreement with them. In ARDS patients with reduced lung volume and inhomogeneity, reducing flow amplitude by increasing T_slope_ and providing a low peak flow may be advantageous (20). Importantly, it was observed that prolonging T_slope_ up to 20% did not induce intrinsic PEEP in this patient population. Additionally, a minimal increase in carbon dioxide, which was not clinically significant, was deemed acceptable. Each 5% increase in T_slope_ resulted in a minimal end-tidal carbon dioxide increase of approximately 1 mmHg and a decrease in tidal volume of approximately 10 ml, which was not clinically relevant (see Additional file 1: Table S4). Longer T_slope_ times resulted in less energy being applied to the respiratory system. High energy levels have been linked to significant lung damage in experimental and clinical studies (over 12 and 17 J/min for ARDS and non-ARDS, respectively) (19, 21–23). It has been suggested that damage increases exponentially as mechanical power exceeds these threshold values due to factors such as gradual breaking of carrier microstructure elements, decreased ventilated areas (baby lungs), increased load on the remaining structures, and the catalytic effect of stress increasers (22).

A notable finding is that WOBv decreased significantly as T_slope_ increased. It is known that WOB decreases when the inspiratory gas flow is reduced in PCV mode, while in VCV mode, increasing gas flow by 20% results in a 37% increase in mechanical power (2, 24, 25). The gas flow profile and high gas flow amplitude have been associated with increased VILI, highlighting the importance of flow pattern and rate in ARDS patients (26–28). Similar findings have been demonstrated in animal models of lung injury (29, 30). Notably, in PCV, mechanical power is concentrated at the beginning of inspiration, while in VCV, it is evenly distributed throughout inspiration (31). This study indicates that increasing T_slope_ and slowing gas flow during the initial phase of inspiration can lead to lower mechanical power applied to the lung and reduced power concentration at the start of inspiration (approximately 1 J/min for every 5% increase).

A recent in-silico study in single and multi-compartment lung models highlighted the importance of flow profile and amplitude in determining strain and strain-related power distribution (32). These findings support the idea that setting longer T_slope_ times to reach the target pressure (∆P_insp_) in pressure control ventilation can provide slower inspiratory gas flow to the lungs during early inspiration, resulting in safer lung ventilation (i.e., less power) (20).

The debate over whether rapid transmission of per-cycle energy to the lungs during early inspiration may increase damage in ARDS patients is ongoing. It is suggested that the descelerating flow pattern may put PCV mode at a disadvantage in this context (33). However, with extended T_slope_ periods in which inspiratory time is adjusted to achieve the target tidal volume (4–6 ml/kg), this concern is alleviated, and lower mechanical power is applied to the lungs. For existing mechanical ventilator brands that display parameters such as work of breathing (WOB) and work of breathing ventilatory (WOBv), the MP_dyn_ equation can be considered as an alternative method for continuously and in real-time calculating mechanical power in control ventilation (PCV) modes without the need for inspiratory resistance measurements.

### Limitations

The results of this study cannot be extrapolated to patients with COPD, who typically exhibit higher median inspiratory resistance values, as our calculations were based on a constant inspiratory resistance value of 15 cmH_2_O s/L. Furthermore, in patients with a stiff chest wall, such as those who are obese or kyphotic, the utilization of WOB and WOB_v_ parameters to estimate the force applied to the lung may introduce a higher margin of error (16). It's worth noting that this limitation applies to all power equations that rely on respiratory parameters obtained from mechanical ventilators. The predictive accuracy of the MP_dyn_ equation might be less apparent to the user for prospective estimation because the equation relies on WOBv calculated by the ventilator and does not incorporate user-controlled variables such as ∆P_insp_ (change in inspiratory pressure) and TVe (tidal volume). Unfortunately, due to the unavailability of the necessary equipment, we were unable to compare the mechanical power values calculated with all the equations against the geometric method, which is widely regarded as the gold standard for power calculation.

## Conclusion

The disparity between mechanical power values computed using the MP_dyn_ and MP_LM_ equations at varying T_slope_ durations did not yield clinically significant differences. We recommend for the use of the MP_dyn_ equation as a practical and continuous alternative for calculating mechanical power in PCV mode. Notably, a substantial reduction in mechanical power was observed with longer T_slope_ durations. Specifically, each 5% increment in T_slope_ corresponded to a decrease in mechanical power by approximately 1 J/min.

In the quest for safer ventilation strategies, inspiratory rise time should be considered as a variable that exerts a clinically significant impact on mechanical power.

### Supplementary Information

**Additional file 1: Table S1.** Mechanical power equations in PCV,VCV, and PCV: Pressure Control Ventilation; MP_pcv_: alternative pressure control power equation, MP_pcv(m-simpl)_: modified pressure control simplified power equation, MP_pcv(simpl)_: simplified pressure control power equation, MP_pcv(slope)_: comprehensive pressure control simplified power equation, VCV: Volume Control Ventilation; MP_std_: standart volume control power equation MP_vcv-simpl_: Gattinoni simplified equation MP_vcv-surr_: volume control surrogate power equation, MP_dyn_: dynamic power equation, MP_pmean_: mean airway pressure (P_mean_) derived power equation, CF: conversion factor. **Figure S1.** Flow chart C-ARDS, Covid-19 related acute respiratory distress syndrome; CHF, congestive heart failure; COPD, Chronic obstructive pulmonary disease; IMV, invasive mechanical ventilation; ECMO, extracorporeal membrane oxygenation; ICU, intensive care unit; LOS, length of stay. **Figure S2.** Dynamic pressure–volume loop (P–V), pressure—time, flow—time and volume—time graphs of IE 1:1 and T_slope_ 5%, 10%, 15% and 20% times of the patient ventilated in passive pressure control mode (obtained with screen shots of the Servo-U mechanical ventilator). **Figure S3.** In univariable logistic regression analyses on mechanical power of all patients average. For IE 1:2 and T-slope 5%: A. MP_LM_ (y-axis) is plotted as function of the MP_dyn_ equation (x-axis). For IE 1:2 and T-slope 10%: B. MP_LM_ (y-axis) is plotted as function of the MP_dyn_ equation (x-axis). For IE 1:2 and T_slope_ 15%: C. MP_LM_ (y-axis) is plotted as function of the MP_dyn_ equation (x-axis). For IE 1:2 and T_slope_ 20%: D. MP_LM_ (y-axis) is plotted as function of the MP_dyn_ equation (x-axis). **Figure S4.** In univariable logistic regression analyses on mechanical power of all patients average. For IE 1:1 and T-slope 5%: A. MP_LM_ (y-axis) is plotted as function of the MP_dyn_ equation (x-axis). For IE 1:1 and T_slope_ 10%: B. MP_LM_ (y-axis) is plotted as function of the MP_dyn_ equation (x-axis). For IE 1:1 and T_slope_ 15%: C. MP_LM_ (y-axis) is plotted as function of the MP_dyn_ equation (x-axis). For IE 1:1 and T-slope 20%: D. MP_LM_ (y-axis) is plotted as function of the MP_dyn_ equation (x-axis). **Table S2.** The patient averages of mechanical power values calculated with MP_LM_ and MP_dyn_ equations at I:E 1:2 and 1:1 ratios at T_slope_ 5%, 10%, 15%, 20% times were compared with the repeated measures MANOVA method. Tukey's multiple comparisons test was used to detect significant difference between groups. **Table S3.** The patient averages of T_*slope*_ 5%, 10%, 15%, and 20% P_insp_, PEEP, MVe and WOBv parameters were compared with the repeated MANOVA method. Tukey's multiple comparisons test was used to detect significant difference between groups. **Table S4.** The patient averages of T_*slope*_ 5%, 10%, 15%, and 20% RR, TVe, T_insp_, Vee, FiO_2_, SpO_2_ and etCO_2_ parameters were compared with the repeated MANOVA method. Tukey's multiple comparisons test was used to detect significant difference between groups.

## Data Availability

The datasets used and/or analysed during the current study are available from the corresponding author on reasonable request.
