# Supplementary material for: The effect of inspiratory rise time on mechanical power calculations in pressure control ventilation: dynamic approach
Source: Intensive Care Med Exp. 2023 Dec 20;11:98. doi: 10.1186/s40635-023-00584-6 (PMC10733269; doi:10.1186/s40635-023-00584-6)
Supplement: Supplementary file 1 — Additional file 1: Table S1. Mechanical power equations in PCV,VCV, and PCV: Pressure Control Ventilation; MPpcv: alternative pressure control power equation, MPpcv(m-simpl): modified pressure control simplified power equation, MPpcv(simpl): simplified pressure control power equation, MPpcv(slope): comprehensive pressure control simplified power equation, VCV: Volume Control Ventilation; MPstd: standart volume control power equation MPvcv-simpl: Gattinoni simplified equation MPvcv-surr: volume control surrogate power equation, MPdyn: dynamic power equation, MPpmean: mean airway pressure (Pmean) derived power equation, CF: conversion factor. Figure S1. Flow chart C-ARDS, Covid-19 related acute respiratory distress syndrome; CHF, congestive heart failure; COPD, Chronic obstructive pulmonary disease; IMV, invasive mechanical ventilation; ECMO, extracorporeal membrane oxygenation; ICU, intensive care unit; LOS, length of stay. Figure S2. Dynamic pressure–volume loop (P–V), pressure—time, flow—time and volume—time graphs of IE 1:1 and Tslope 5%, 10%, 15% and 20% times of the patient ventilated in passive pressure control mode (obtained with screen shots of the Servo-U mechanical ventilator). Figure S3. In univariable logistic regression analyses on mechanical power of all patients average. For IE 1:2 and T-slope 5%: A. MPLM (y-axis) is plotted as function of the MPdyn equation (x-axis). For IE 1:2 and T-slope 10%: B. MPLM (y-axis) is plotted as function of the MPdyn equation (x-axis). For IE 1:2 and Tslope 15%: C. MPLM (y-axis) is plotted as function of the MPdyn equation (x-axis). For IE 1:2 and Tslope 20%: D. MPLM (y-axis) is plotted as function of the MPdyn equation (x-axis). Figure S4. In univariable logistic regression analyses on mechanical power of all patients average. For IE 1:1 and T-slope 5%: A. MPLM (y-axis) is plotted as function of the MPdyn equation (x-axis). For IE 1:1 and Tslope 10%: B. MPLM (y-axis) is plotted as function of the MPdyn equation (x-axis). [file 40635_2023_584_MOESM1_ESM.docx]

**Additional file**

| **Power**  **equations** | **Authors** |  |
| --- | --- | --- |
| **PCV** |  |  |
| **MP_pcv_ ^1^** | Van der Meijden et al | 0,098 x RR x ∆V x [PEEP + ∆P_insp_ x (1 – e ^– Tins^ **^/^** ^(R x C)^)] |
| **MP_pcv(m-simpl)_ ^2^** | Aşar et al | 0,098 x RR x ∆V x (PEEP + ∆_Pinsp_ -1) |
| **MP_pcv(slope)_ ^3^** | Becher et al | 0,098 x RR x [(∆P_insp_ + PEEP) x ∆V - ∆P_insp_^2^ x C x (0.5 – (R x C)/T_slope_ + (R x C/ T_slope_)^2^ x (1 – e ^– Tslope/ (R x C^))] |
| **MP_pcv(simpl)_ ^3^** | Becher et al | 0,098 x RR x ∆V x (PEEP + ∆P_insp_ ) |
| **MP_LM_ ^4^** | Trinkle et al | 0,098 x RR x [∆V x (PEEP + ∆P_insp_) -0.15 x ∆P_insp_^2^ x T_slope_/R |
| **VCV** |  |  |
| **MP_std_ ^5^** | Gattinoni et al | 0,098 x RR x {∆V^2^ [0,5 x Elrs + RR x Raw x (1+I:E)/60 x I:E)] + ∆V x PEEP} |
| **MP_vcv-simpl_ ^5^** | Gattinoni et al | 0,098 x RR x ∆V x (P_peak_-DP/2) |
| **MP_surr_ ^6^** | Giosa et all | MVe (P_peak_+PEEP+F/6)/20 |
| **MP_dyn_ ^7^** | Aşar et al | MVe x [(WOBv)+ (PEEP x 0.098)] |
| **MP_pmean_ ^8^** | Yi Chi et al | 0.1 x ∆V x[P_mean_+ (P_mean_-PEEP)x Te/Ti)] |

**Table 1S.** Mechanical power equations in PCV ,VCV, and PCV: Pressure Control Ventilation; MP_pcv_: alternative pressure control power equation, MP_pcv(m-simpl)_: modified pressure control simplified power equation, MP_pcv(simpl)_: simplified pressure control power equation, MP_pcv(slope)_: comprehensive pressure control simplified power equation, VCV : Volume Control Ventilation ; MP_std_ : standart volume control power equation MP_vcv-simpl_: Gattinoni simplified equation MP_vcv-surr_: volume control surrogate power equation, MP_dyn_: dynamic power equation , MP_pmean_: mean airway pressure (P_mean_) derived power equation, CF: conversion factor

**Figure 1S:** Flow chart C-ARDS, Covid-19 related acute respiratory distress syndrome; CHF, congestive heart failure; COPD, Chronic obstructive pulmonary disease; IMV, invasive mechanical ventilation; ECMO, extracorporeal membrane oxygenation; ICU, intensive care unit; LOS, length of stay.

**
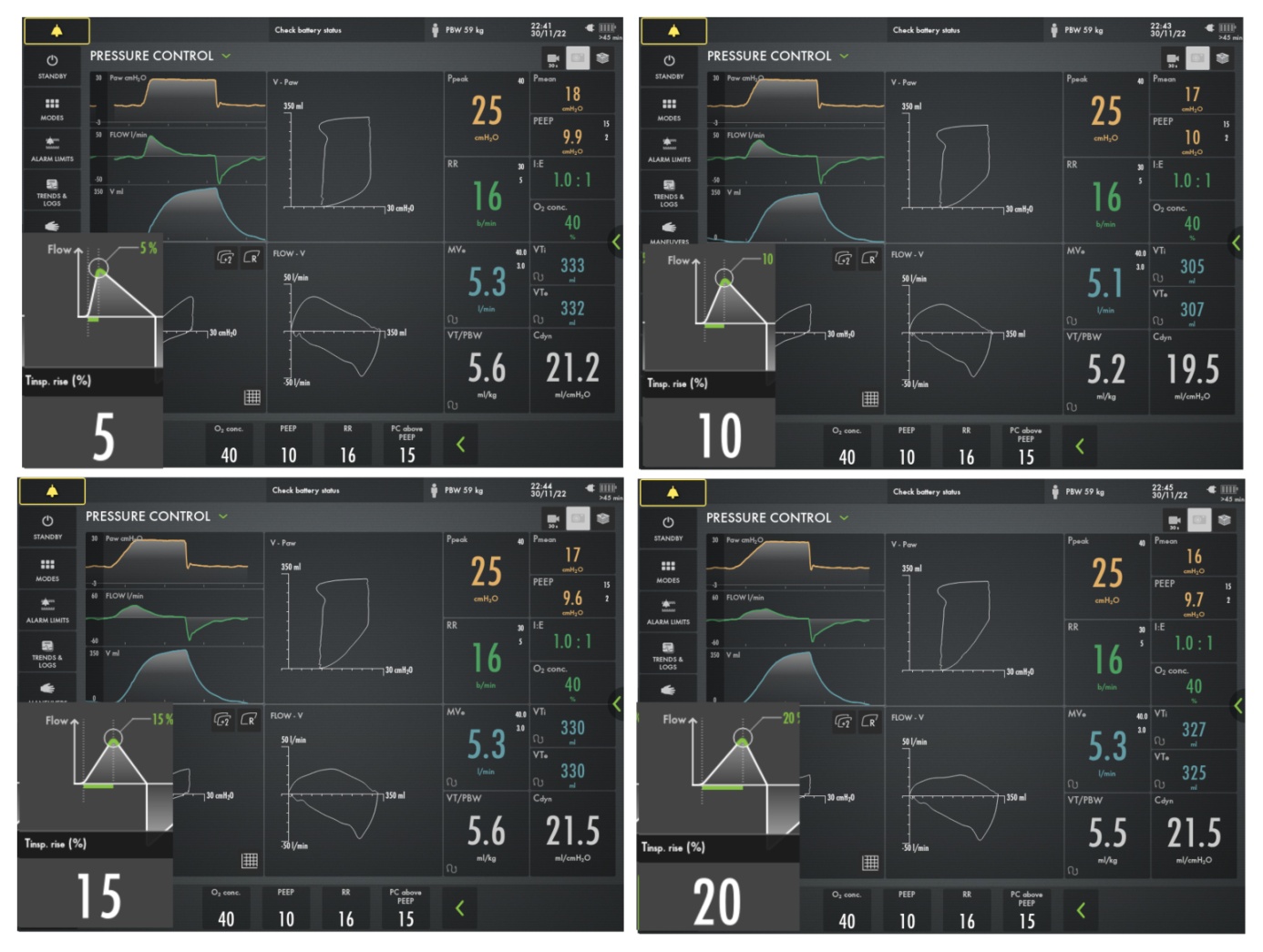
**

**Figure 2S.** Dynamic pressure-volume loop (P-V), pressure - time, flow - time and volume - time graphs of IE 1:1 and T_slope_ 5%, 10%, 15% and 20% times of the patient ventilated in passive pressure control mode (obtained with screen shots of the Servo-U mechanical ventilator).

**
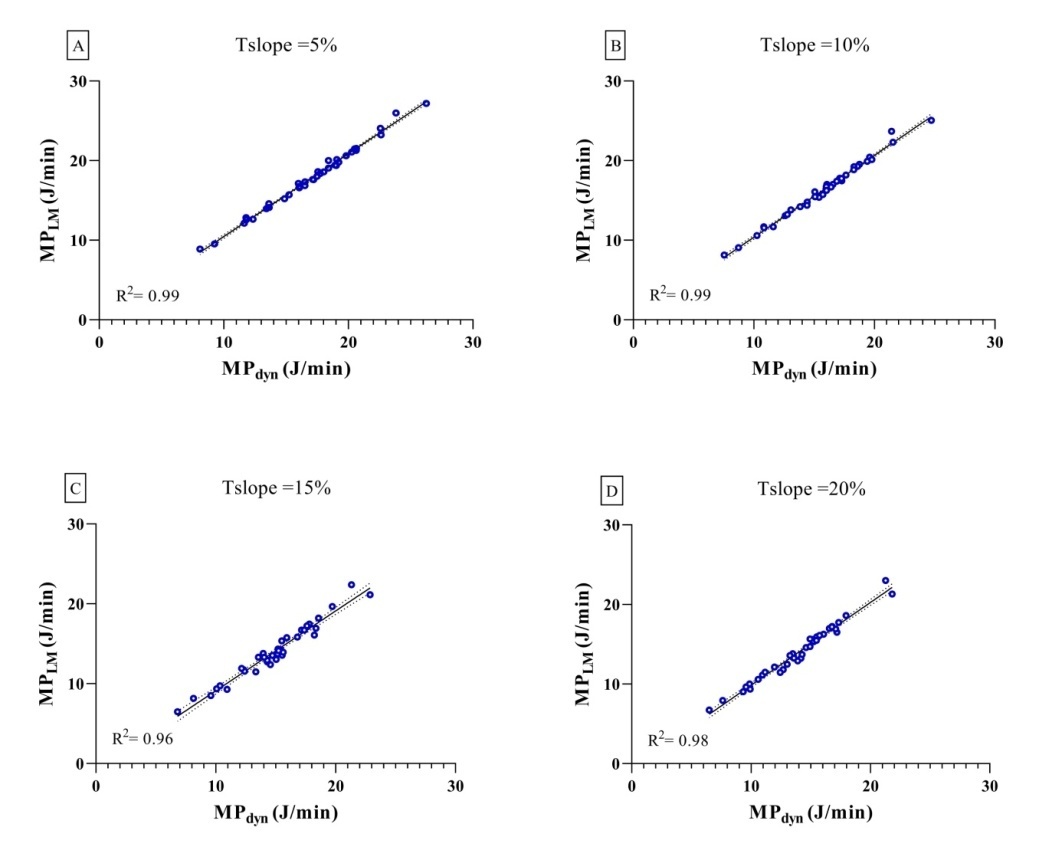
**

**Figure 3S.** In univariable logistic regression analyses on mechanical power of all patients average. For IE 1:2 and T-slope 5%: A. MP_LM_ (y-axis) is plotted as function of the MP_dyn_ equation (x-axis). For IE 1:2 and T-slope 10%: B. MP_LM_ (y-axis) is plotted as function of the MP_dyn_ equation (x-axis). For IE 1:2 and T_slope_ 15%: C. MP_LM_ (y-axis) is plotted as function of the MP_dyn_ equation (x-axis). For IE 1:2 and T_slope_ 20%: D. MP_LM_ (y-axis) is plotted as function of the MP_dyn_ equation (x-axis).


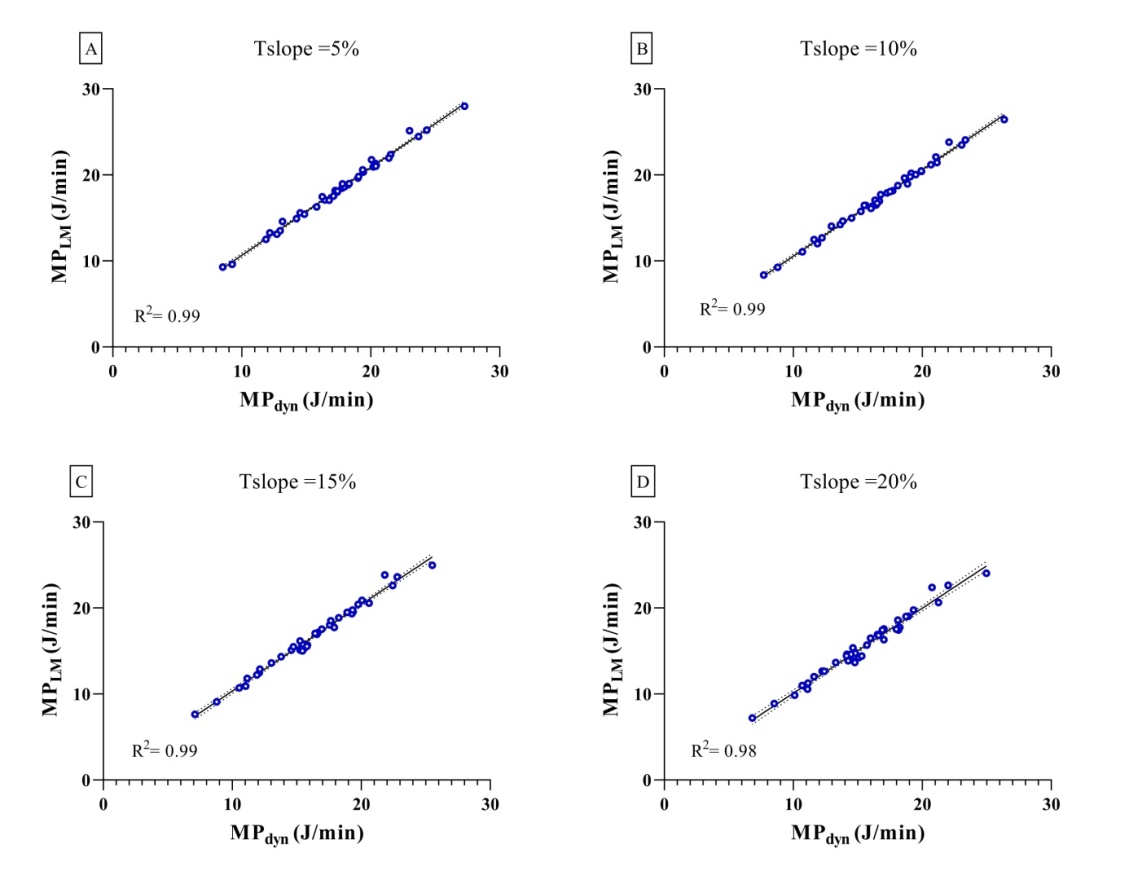


**Figure 4S.** In univariable logistic regression analyses on mechanical power of all patients average. For IE 1:1 and T-slope 5%: A. MP_LM_ (y-axis) is plotted as function of the MP_dyn_ equation (x-axis). For IE 1:1 and T_slope_ 10%: B. MP_LM_ (y-axis) is plotted as function of the MP_dyn_ equation (x-axis). For IE 1:1 and T_slope_ 15%: C. MP_LM_ (y-axis) is plotted as function of the MP_dyn_ equation (x-axis). For IE 1:1 and T-slope 20%: D. MP_LM_ (y-axis) is plotted as function of the MP_dyn_ equation (x-axis).

**Repeated MANOVA analysis*:***

|  | **IE 1:2 ratio** | | **IE 1:1 ratio** | |
| --- | --- | --- | --- | --- |
|  | **mean of difference (CI)** | **p-value** | **mean of difference (CI)** | **p-value** |
| **MP_LM_, (J/min)** |  |  |  |  |
| *T_slope_ 5% vs 10%* | 1,36 (0,93 to 1,79) | < .0001 | 0.90 (0.71 to 1.09) | < .0001 |
| *T_slope_10% vs 15%* | 1,14 (0,82 to 1,46) | < .0001 | 0.87 (0.68 to 1.07) | < .0001 |
| *T_slope_15% vs 20%* | 1,23 (0,92 to 1,54) | < .0001 | 1.11 (0.81 to 1.41) | < .0001 |
| **MP_dyn_, (J/min)** |  |  |  |  |
| *T_slope_5% vs 10%* | 1.16 (0.79 to 1.52) | < .0001 | 0.68 (0.54 to 0.83) | < .0001 |
| *T_slope_10% vs 15%* | 0.90 (0.64 to 1.16) | < .0001 | 0.66 (0.50 to 0.82) | < .0001 |
| *T_slope_15% vs 20%* | 0.93 (0.65 to 1.21) | < .0001 | 0.76 (0.52 to 0.99) | < .0001 |

**Table 2S.** The patient averages of mechanical power values calculated with MP_LM_ and MP_dyn_ equations at I:E 1:2 and 1:1 ratios at T_slope_ 5%, 10%, 15%, 20% times were compared with the repeated measures MANOVA method. Tukey's multiple comparisons test was used to detect significant difference between groups.

Mean mechanical power calculated for T_slope_ 5%, 10%, 15% and 20% of MP_LM_ and MP_dyn_ equations at IE 1:2 and I:E 1:1 ratios was calculated with T_slope_ 5% versus 10%, T_slope_ 10% versus 15% and T_slope_ 15%versus 20%, mean of difference and confident interval (CI) values were evaluated as statistically significant (< .0001). For every 5% increase in T_slope_ time at I:E 1:2 ratio, approximately 1.2 J/min decrease in mechanical power values calculated with MP_LM_ equation, and approximately 1.0 J/min decrease for MP_dyn_ was detected (p<0.0001, Table 2S). Likewise, for every 5% increase in T_slope_ time for I:E 1:1 ratio, approximately 1.0 J/min decrease in mechanical power values calculated with MP_LM_ equation, and approximately 0.7 J/min decrease for MP_dyn_ was determined (p<0.0001, Table 2S).

Mean and standard deviation values of mechanical power patient averages calculated for T_slope_ 5%, 10%, 15% and 20% with MP_dyn_ equation at I:E 1:2 ratio, was calculated as 16.9 ± 3.8 J/min, 15.7 ± 3.5 J/min, 14.9 ± 3.3 J/min ,13.9 ± 3.2 J/min respectively. For every 5% increase in the T_slope_ time, approximately 1.0 J/min decrease was detected in the mechanical power values calculated with the MP_LM_ equation (p<0.0001, Table 2S). Mean of differences, confident interval (CI) and p-values of mechanical power averages calculated with MP_LM_ and MP_dyn_ equations at IE 1:2 and IE 1:1 ratios comparison with T_slope_ 5% versus 10%, 10% versus 15% and 15% versus 20% are shown in Table 2S.

**Repeated MANOVA *analysis:***

| **The average of all patients parameters (n= 38)** | **IE 1:2 ratio** | | **IE 1:1 ratio** | |
| --- | --- | --- | --- | --- |
|  | **mean of difference (CI)** | **p-value** | **mean of difference (CI)** | **p-value** |
| **∆P_insp_, cmH_2_O** |  |  |  |  |
| *T_slope_ 5% vs 10%* | 16,31 vs 16,32 | 0.7 | 16,31 vs 16,32 | 0.7 |
| *T_slope_ 10% vs 15%* | 16,32 vs 16,32 | >0,9999 | 16,32 vs 16,32 | >0,9999 |
| *T_slope_ 15% vs 20%* | 16,32 vs 16,32 | >0,9999 | 16,32 vs 16,32 | >0,9999 |
| **PEEP, cmH_2_O** |  |  |  |  |
| *T_slope_ 5% vs 10%* | 9.01 vs 9.03 | 0.7 | 9.18 vs 9.18 | 0.82 |
| *T_slope_ 10% vs 15%* | 9.03 vs 9.0 | 0.06 | 9.18 vs 9.18 | >0,99 |
| *T_slope_ 15% vs 20%* | 9.0 vs 9.01 | >0,9999 | 9.18 vs 9.17 | 0.88 |
| **T_slope_, second** |  |  |  |  |
| *T_slope_ 5% vs 10%* | 0.184 vs 0.367 | <0,0001 | 0.184 vs 0.367 | <0,0001 |
| *T_slope_ 10% vs 15%* | 0.367 vs 0.547 | <0,0001 | 0.367 vs 0.547 | <0,0001 |
| *T_slope_ 15% vs 20%* | 0.547 vs 0.732 | <0,0001 | 0.547 vs 0.732 | <0,0001 |
| **MVe, L** |  |  |  |  |
| *T_slope_ 5% vs 10%* | 7.44 vs 7.20 | 0,0017 | 7.64 vs 7.59 | 0.07 |
| *T_slope_ 10% vs 15%* | 7.20 vs 7.07 | 0,0211 | 7.59 vs 7.55 | 0.47 |
| *T_slope_ 15% vs 20%* | 7.07 vs 6.90 | 0,0056 | 7.55 vs 7.45 | 0,03 |
| **WOBv, J** |  |  |  |  |
| *T_slope_ 5% vs 10%* | 1.39 vs 1.30 | <0,0001 | 1.38 vs 1.31 | <0,0001 |
| *T_slope_ 10% vs 15%* | 1.30 vs 1.21 | <0,0001 | 1.31 vs 1.23 | <0,0001 |
| *T_slope_ 15% vs 20%* | 1.21 vs 1.130 | <0,0001 | 1.23 vs 1.16 | <0,0001 |

**Table 3S.** The patient averages of T*_slope_* 5%, 10%, 15%, and 20% P_insp_, PEEP, MVe and WOBv parameters were compared with the repeated MANOVA method. Tukey's multiple comparisons test was used to detect significant difference between groups.

Respiratory mechanics, P_insp_, PEEP, MVe, WOBv parameters that contribute to the mechanical power calculation of MP_LM_ and MP_dyn_ formulas, measured patient averages for T_slope_ 5%, 10%, 15% and 20%, T*_slope_* 5% vs 10%, T*_slope_* 10% vs 15%, and T-slope of 15% vs 20% was compared with repeated MANOVA test. The mean of difference and confident interval (CI) values of P_insp_ and PEEP parameters (set as fixed) of comparisons of T*_slope_* 5% vs 10%, T_slope_ 10% vs 15% and T*_slope_*15% vs 20% at IE 1:2 and IE 1:1 ratios difference were not considered statistically significant (p> .017). For every 5% increase in IE 1:2 T-slope time, approximately 0.18 L decrease was detected in MVe values on average (p values 0.0017, 0.0211, 0.0056, respectively, Table 4S). For every 5% increase in T_slope_ time of IE 1:1 ratio of MVe, approximately 0.06 L decrease was detected on average, and the differences were not evaluated statistically significant (p>.017, Table 4S).IE 1:2 ratio T_slope_ A 0.09J decrease in WOBv values was determined for every 5% increase in duration (<0.0001, Table 4S). At IE 1:1 ratio, an average of 0.07 J decrease was found in WOBv values for every 5% increase in T*_slope_* time in all three comparisons (p<0.0001, Table 4S). The mean of differences, CI of P_insp_, PEEP, T_slope_ MVe and WOBv parameters of the patient averages calculated at IE 1:2 and IE 1:1 ratios, T_slope_ 5% vs 10%, 10% vs 15% and 15% vs 20% comparisons. CI and p values are shown in Table 4S.

**Repeated MANOVA analysis:**

| **Respiratory mechanics** | **5% (n=38)** | **10% (n=38)** | **15% (n=38)** | **20% (n=38)** | **P value** |
| --- | --- | --- | --- | --- | --- |
|  | **mean ± sd** | **mean ± sd** | **mean ± sd** | **mean ± sd** |  |
| TVe, ml, IE 1:2  IE 1:1 | 448 ± 71 | 440 ± 71 | 431 ± 69 | 420 ± 66 | <0.0001 |
|  | 465 ± 75 | 463 ± 75 | 460 ± 77 | 455 ± 76 | <0.0001 |
| C, ml/cmH_2_O, IE 1:2  IE 1:1 | 30.7 ± 8.0 | 30.4 ± 7.8 | 30.3 ± 7.8 | 30.1 ± 8.0 | 0.0002 |
|  | 30.7 ± 8.0 | 30.4 ± 7.8 | 30.2 ± 7.8 | 30.1 ± 8.0 | 0.0002 |
| RR 1/min, IE 1:2  IE 1:1 | 16.6 ± 1.8 | 16.5 ± 1.7 | 16.5 ± 1.7 | 16.5 ± 1.8 | 0.1 |
|  | 16.5 ± 1.8 | 16.5 ± 1.7 | 16.5 ± 1.8 | 16.5 ± 1.7 | 0.7 |
| T_insp_, sec, IE 1:2  IE 1:1 | 1.22 ± 0.12 | 1.22 ± 0.12 | 1.22 ± 0.12 | 1.22 ± 0.12 | 0.3 |
|  | 1.83 ± 0.18 | 1.83 ± 0.18 | 1.83 ± 0.18 | 1.83 ± 0.18 | 0.9 |
| Vee, L/sec, IE 1:2  IE 1:1 | 0.014 ± 0.01 | 0.014 ± 0.01 | 0.013 ± 0.01 | 0.012 ± 0.01 | 0.0006 |
|  | 0.034 ± 0.02 | 0.034 ± 0.02 | 0.035 ± 0.02 | 0.033 ± 0.02 | 0.4 |
| SpO_2_, IE 1:2  IE 1:1 | 95 ± 2 | 95 ± 2 | 95 ± 3 | 95 ± 3 | 0.3 |
|  | 95 ± 3 | 95 ± 3 | 95 ± 3 | 95 ± 2 | 0.2 |
| etCO_2_, IE 1:2  IE 1:1 | 55 ± 13 | 57 ± 13 | 58 ± 13 | 59 ± 14 | 0.008 |
|  | 50 ± 9 | 51 ± 10 | 52 ± 10 | 54 ± 11 | 0.0001 |

**Table 4S.** The patient averages of T*_slope_* 5%, 10%, 15%, and 20% RR, TVe, T_insp_, Vee, FiO_2_, SpO_2_ and etCO_2_ parameters were compared with the repeated MANOVA method. Tukey's multiple comparisons test was used to detect significant difference between groups.

Patient averages of 20-minute measurements of respiratory mechanics (RR, PEEP, TVe, P_insp_, T*_slope_* time) in which mechanical power is calculated at IE 1:2 and 1:1 ratios, T*_slope_*5%, 10%, 15%, and 20% values of patients, compared with the repeated MANOVA method. Tukey's multiple comparisons test was performed to detect significant difference between groups. Similar analysis was performed for WOBv, Vee, FiO_2_, SpO_2_ and etCO_2_ parameters measured at the same time periods. A statistically significant difference was found between the TVe, Clung, WOBv, and etCO_2_ values of the patients' T*_slope_* values at IE 1:2 and 1:1 ratios (p<0.017). There was no statistically significant difference in P_insp_, PEEP, RR and SpO_2_ values of both IE ratios. A statistically significant difference was found between different T*_slope_* Vee values of IE 1:2 ratio, but no statistically significant difference was found between different T*_slope_* Vee values of IE 1:1 ratio. The mean, standard deviation and p values of the T*_slope_* values of all parameters are shown in Table 3S.

**References:**

1. Van der Meijden S, Molenaar M, Somhorst P, Schoe A. Calculating mechanical power for pressure-controlled ventilation. Intensive Care Med 2019;45:1495–7. 7.
2. Aşar, Sinan, et al. "Simplified calculation of mechanical power for pressure controlled ventilation in Covid-19 ARDS patients." *Minerva Anestesiologica* 88.1-2 (2022): 42-50.
3. Becher T, van der Staay M, Schädler D, Frerichs I, Weiler N. Calculation of mechanical power for pressure-controlled ventilation. Intensive Care Med 2019;45:1321–3. 6.
4. Trinkle, Christine A., et al. "Simple, accurate calculation of mechanical power in pressure controlled ventilation (PCV)." *Intensive Care Medicine Experimental* 10.1 (2022): 1-12.
5. Gattinoni L, Tonetti T, Cressoni M, Cadringher P, Herrmann P, Moerer O, et al. Ventilator-related causes of lung injury: the mechanical power. Intensive Care Med 2016;42:1567–75.
6. Giosa, Lorenzo, et al. "Mechanical power at a glance: a simple surrogate for volume-controlled ventilation." *Intensive care medicine experimental* 7.1 (2019): 1-13.
7. Aşar, Sinan, et al. "Bedside dynamic calculation of mechanical power: A validation study." *Journal of Critical Care* 56 (2020): 167-170.
8. Chi, Yi, Huaiwu He, and Yun Long. "A simple method of mechanical power calculation: using mean airway pressure to replace plateau pressure." *Journal of Clinical Monitoring and Computing* 35.5 (2021): 1139-1147.

**Mechanical power equations software codes:**

Clinical Decision Support Software (ImdSoft-Metavision/QlinICU) used on a mechanical ventilator breathing parameters minute data recorded every minute of patients power equations (MP_dyn_ and MP_LM_)) is applied by converting the computer software program as shown below. Each measurement corresponds to one minute.

**MP_dyn__equation**

If Parameters.UValue("MVe")>0 Then

mve=(Parameters.UValue("MVe"))

Else

mve=0

End IF

If Parameters.UValue("Peep")>0 Then

peep=Parameters.UValue("Peep")

Else

peep=0

End IF

If Parameters.UValue("WOBv")>0 Then

w=(Parameters.UValue("WOBv"))

Else

w=0

End IF

If (w>0) and (mve>0) and (peep>0) then

p=Round(((w*mve)+(peep*mve*0.098)),2)

Else

p="--"

End If

Return_Value=p

**MP_LM_ equation**

If Parameters.UValue("MVe")>0 and Parameters.UValue("TVe")>0 Then

MVe=Parameters.UValue("RR")* Parameters.UValue("TVe")

Else

MVe=0

End IF

If Parameters.UValue("Peep")>0 Then

peep=Parameters.UValue("Peep")

Else

peep=0

End IF

If Parameters.UValue("Pinsp ")>0 Then

Pinsp =Parameters.UValue("Pinsp ")

Else

P_insp_ =0

End IF

If Parameters.UValue("Tslope")>0 Then

Tslope_t=(Parameters.UValue("Tslope"))

Else

T_slope__t =0

End IF

If (P_insp_ >0) and (mve>0) and (peep>0) and (T_slope__t) then

p=Round((((mve*0.098)*(peep+ Pinsp)- 0.15* Pinsp* Pinsp* Tslope_t/15))),2

Else

p="--"

End If

Return_Value=p
